# Integrating lipidomics and genomics: emerging tools to understand cardiovascular diseases

**DOI:** 10.1007/s00018-020-03715-4

**Published:** 2021-01-15

**Authors:** Rubina Tabassum, Samuli Ripatti

**Affiliations:** 1 Institute for Molecular Medicine Finland (FIMM), HiLIFE, University of Helsinki, PO Box 20, 00014 Helsinki, Finland; 2 Department of Public Health, Clinicum, University of Helsinki, Helsinki, Finland; 3 Broad Institute of the Massachusetts Institute of Technology and Harvard, Cambridge, MA USA

**Keywords:** Lipid species, Biomarkers, Genome-wide association studies, Disease risk, Pathophysiology

## Abstract

Cardiovascular diseases (CVDs) are the leading cause of mortality and morbidity worldwide leading to 31% of all global deaths. Early prediction and prevention could greatly reduce the enormous socio-economic burden posed by CVDs. Plasma lipids have been at the center stage of the prediction and prevention strategies for CVDs that have mostly relied on traditional lipids (total cholesterol, total triglycerides, HDL-C and LDL-C). The tremendous advancement in the field of lipidomics in last two decades has facilitated the research efforts to unravel the metabolic dysregulation in CVDs and their genetic determinants, enabling the understanding of pathophysiological mechanisms and identification of predictive biomarkers, beyond traditional lipids. This review presents an overview of the application of lipidomics in epidemiological and genetic studies and their contributions to the current understanding of the field. We review findings of these studies and discuss examples that demonstrates the potential of lipidomics in revealing new biology not captured by traditional lipids and lipoprotein measurements. The promising findings from these studies have raised new opportunities in the fields of personalized and predictive medicine for CVDs. The review further discusses prospects of integrating emerging genomics tools with the high-dimensional lipidome to move forward from the statistical associations towards biological understanding, therapeutic target development and risk prediction. We believe that integrating genomics with lipidome holds a great potential but further advancements in statistical and computational tools are needed to handle the high-dimensional and correlated lipidome.

**Supplementary Information:**

The online version contains supplementary material available at 10.1007/s00018-020-03715-4.

## Introduction

**Fig. 1:**
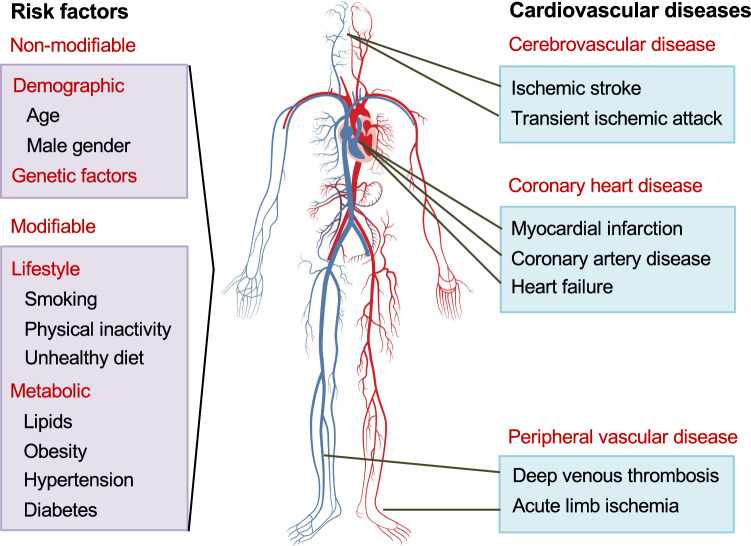
Cardiovascular diseases and their risk factors. CVDs encompass a broad range of disorders affecting the heart, brain and blood vessels. The different manifestations of CVDs include myocardial infarction, stroke and peripheral artery disease. A number of modifiable and non-modifiable risk factors have been identified that predispose individuals to CVDs. Relationships between lifestyle factors and lipids are well known and have been the target for prevention strategies

Cardiovascular diseases (CVDs) are a group of complex disorders affecting heart function, vascular structure and circulatory system. Genetic and epidemiological studies have greatly improved our understanding of pathophysiology underlying the complex CVDs and have identified several risk factors for CVDs. Amongst the well-recognized predisposing factors (Fig. 1), lipid metabolism plays a central role in the development of CVDs [1, 2]. Since the landmark publications from the Framingham study [3], plasma lipids have been recognized as important predictors of future CVD events, with lipid lowering as a well-established intervention to reduce CVD risk [4]. To assess CVD risk, plasma lipids are routinely monitored by profiling total cholesterol, triglycerides, high-density lipoprotein cholesterol (HDL-C) and low-density lipoprotein cholesterol (LDL-C) (referred as “traditional lipids”). Despite these advances, CVDs remain the leading cause of mortality and morbidity worldwide [5], as the current preventive strategies are ineffective in a large proportion of the population [6].

Human plasma is estimated to consist of thousands of functionally and chemically diverse molecular lipid species [7–9]. Because of the technological challenges to detect diverse yet structurally similar lipids and their isomers, efforts to understand the role of lipids in CVD pathophysiology had largely focused on traditional lipids, and to some extent on free fatty acids and lipoproteins, until last decade. Nevertheless, there have been tremendous advancements in the field of lipidomics that has facilitated the efforts to unravel the metabolic dysregulation in complex lipid-related disorders, particularly CVDs and to identify predictive biomarkers beyond traditional lipids [10, 11]. The promising findings from epidemiological studies have also led to a growing interest in understanding the genetic regulation of lipid metabolism at molecular lipid species level. Consequently, genome-wide association studies (GWAS) of lipidome profiles, have not only identified new genetic loci/genes influencing distinct molecular species but have also provided novel mechanistic insights to the known genetic loci associated with traditional lipids [12, 13].

This review presents an overview of the application of lipidomics in epidemiological and genetic studies of CVDs and their contributions to the current understanding of the field, along with a brief overview of lipidome diversity and commonly used analytical approaches. The review further discusses some new opportunities provided by integrating emerging genomics tools with the high-dimensional lipidome to move forward from the statistical associations towards therapeutic target development and personalized medicine with better prediction and prevention.

## Human plasma lipidome

Lipidome, the total lipid content in a cell or tissue, is estimated to contain  ~ 200,000 different molecular species with different abundance [9]. This extreme diversity arises from the extraordinary number of possible combinations of various head groups with numerous fatty acids of varying length and degree of unsaturation that are esterified to the head groups. The LIPID MAPS Initiative and the International Committee for the Classification and Nomenclature of Lipids (ICCNL) have provided a standard nomenclature that classifies lipids into eight categories—fatty acyls, glycerolipids, glycerophospholipids, sphingolipids, sterols, prenols, saccharolipids and polyketides [14–16]. Lipids in each lipid category are further divided into classes and subclasses based on the head group and type of linkages [17, https://www.lipidmaps.org/].

**Fig. 2:**
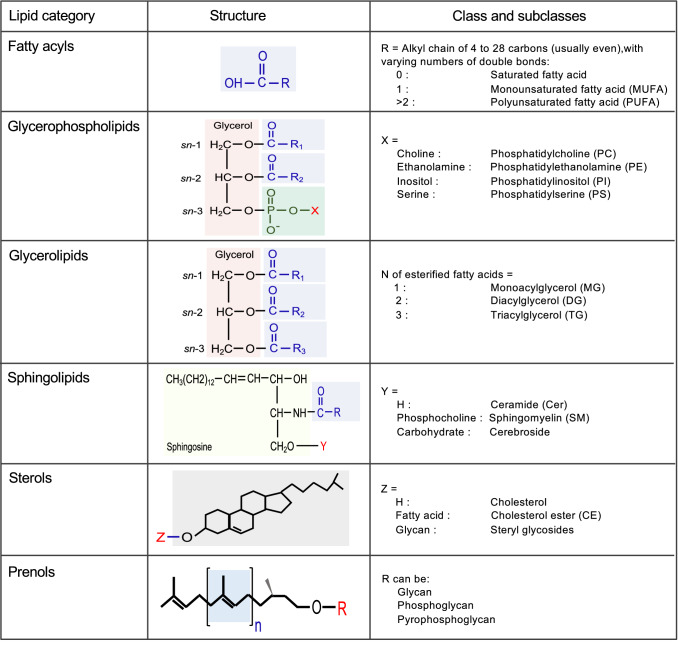
Human plasma lipidome. Six major lipid categories, of eight described by the LIPID MAPS classification system, are illustrated with their classes/subclasses and structure of representative of each lipid category. As shown, esterification of fatty acids with different backbone generates complex lipids including glycerolipids, glycerophospholipids and sphingolipds. Lipids in each lipid category are further divided into classes and subclasses based on the head group and type of linkages between the backbone and acyl chains [https://www.lipidmaps.org/]

The technological advances have tremendously helped in revealing the complexity of lipidome. The LIPID MAPS consortium revealed over 500 molecular lipid species from 6 major lipid categories, with over 200 and 160 distinct species of sphingolipids and glycerophospholipids respectively [18]. A lipidome analysis of human platelets detected over 5,600 unique lipids, with ~ 50% unidentified molecular species [19]. The major lipid categories that are commonly identified in plasma lipidomics are discussed briefly here (Fig. 2), but are reviewed in detail in [7, 15, 18, 20, 21].

### Fatty acyls

Fatty acyls represent the most fundamental category of the lipids including fatty acids. Mostly present in esterified form with glycerol, cholesterol or other lipid components, fatty acids are carboxylic acids, often with long unbranched aliphatic chains of diverse length. Fatty acids are categorized as saturated (no carbon–carbon double bonds in aliphatic chain) and unsaturated with one (monounsaturated fatty acid-MUFA) or more double bonds (polyunsaturated fatty acid-PUFA). Human body can synthesize many of these fatty acids, except some essential fatty acids including linoleic acid (omega-6 PUFA) and alpha-linolenic acid (omega-3 PUFA). These two PUFAs are precursors for other omega-6 and omega-3 PUFAs that play crucial roles in regulating lipid metabolism and atherosclerosis [reviewed in 22, 23].

### Glycerolipids

Esterification of one, two or three fatty acyls to glycerol lead to the formation of glycerolipids and are accordingly classified as monoacylglycerol (MG), diacylglycerol (DG) and triacylglycerol (TG). Glycerolipids are a large group of lipids accounting for a high proportion of total lipids in plasma. TG is the most abundant lipid class and comprises the bulk of storage fat in tissues. MGs and DGs represent intermediates in the biosynthesis and hydrolysis of TGs and function as second messengers in signal transduction processes [24, 25].

### Glycerophospholipids

Also known as phospholipids, glycerophospholipids are diacylglycerides with a phosphatidyl ester attached to the terminal carbon. The terminal ester groups are mainly ethanolamine, choline, serine or inositol (Fig. 2). In addition, a number of fatty acids with varying length and unsaturation could attach to the remaining hydroxyl groups of glycerol via either acyl-, alkyl-, or alkenyl-bonds [18]. Hydrolysis of one of the fatty acids of the phospholipids by phospholipase A2 (PLA2) generates respective lysophospholipids, adding to the diversity of the lipid pool. Glycerophospholipids are the major structural component of cell membranes and are involved in various biological processes including inflammation [21].

### Sphingolipids

**Fig. 3:**
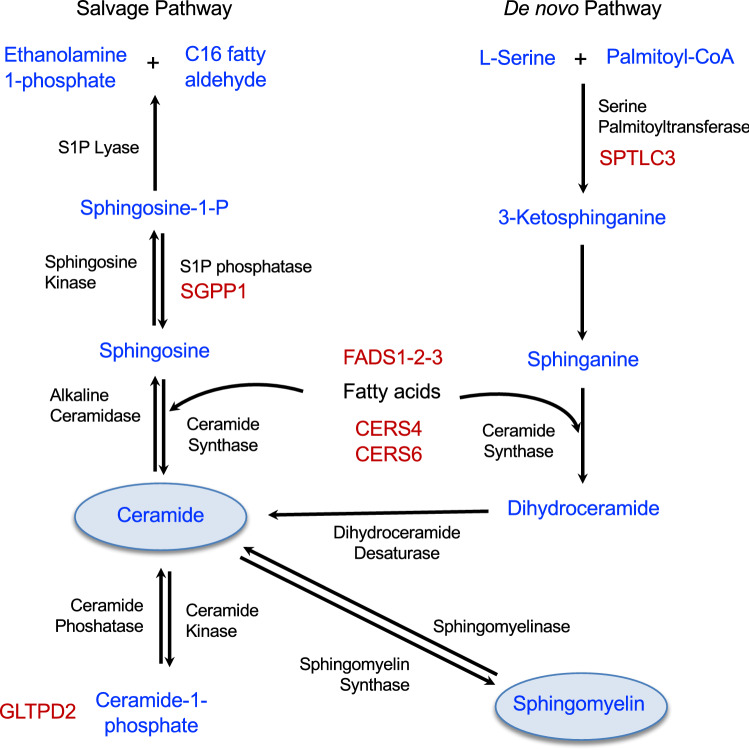
Role of sphingolipid associated loci in major sphingolipid metabolic pathways. Most of the sphingolipid associated loci contain genes that code for enzymes (highlighted in red font) involved in sphingolipid metabolic pathways. SGPP1 codes for a S1P phosphatase that catalyzes the degradation of sphingosine-1-P to sphingosine to facilitate ceramides synthesis catalyzed by ceramide synthases (CERS1-6) including CERS4 and CERS6. *SPTLC3* gene encodes a subunit of the serine palmitoyltransferase complex which catalyzes the rate-limiting step of de novo pathway in sphingolipid biosynthesis. *FADS1-2-3* locus encodes enzymes that regulate the desaturation of fatty acids and have important role in generation of unsaturated ceramides. *GLTPD2* codes for glycolipid transfer protein domain-containing protein 2 and has putative role in transfer of ceramide-1-phosphate

Sphingolipids are wide-range of complex lipids defined by 18-carbon sphingoid base, usually sphingosine (SPH). Condensation of SPH and free fatty acid generates the simplest sphingolipids, ceramides which function as precursor for complex sphingolipids produced by the modification of hydroxyl group with phosphocholine (in sphingomyelins) or carbohydrates (in gangliosides) (Figs. 2, 3) [21, 26]. Sphingolipids constitute several hundreds of different species originating from the combinations of different sphingoid bases, various fatty acids that can attach to the bases and numerous carbohydrates in gangliosides. Ceramide regulates numerous cellular processes such as proliferation, differentiation, and cell signalling [27].

### Sterol lipids

Sterols typically have a sterol nucleus composed of four tightly fused carbon rings and a hydroxyl group attached to the first ring [28]. Cholesterol, the well-known and widely measured lipid, is the simplest and most abundant sterol in plasma, accounting for more than 99% of all plasma sterols. Cholesterol exits in both free and esterified forms as cholesterol esters, mainly in association with lipoproteins.

### Prenols

Prenols are synthesized from five-carbon isoprene units that can be combined in wide variety of polymeric units and configuration to make diverse products [21]. Prenols include vitamins A, E and carotenoids and are essential for immune system or regulatory functions in the brain [21]. Prenols are understudied in the current lipidomic technologies.

## Analytical methods in lipidomics

Of the hundreds of thousands of distinct lipid structures that are estimated to occur in nature, only a small fraction of lipids has been identified so far, highlighting the lack of knowledge and importance of development of high-throughput screening methods for lipid identification and profiling. There are two main approaches in lipidomics: (a) targeted approach that focuses on detection of known lipids using pre-existing knowledge and (b) non-targeted approach that screens all the lipid species without preselection. Non-targeted approach provides large coverage but is limited by the complexity of data processing and identification of lipids from large number of signals. Given the structural diversity of lipid species, no single analytical method could capture the entire lipidome, hence many different methods have been employed [reviewed in 29–35]. Here we briefly discuss two methods—Nuclear Magnetic Resonance (NMR) spectroscopy and Mass spectrometry (MS), that have been used commonly in the epidemiological and genetic studies of lipidome in the context of CVDs.

Lipidome analysis using NMR spectrometry is based on the measurement of magnetic spin of nuclei (^1^H, ^13^C, ^15^N and ^31^P) contained in the lipids. NMR can efficiently and accurately quantify density, size and particle number of different lipoprotein subclasses along with their total lipid content (e.g., total TGs, total phospholipids, total cholesteryl esters, total sphingolipids in HDLs, LDLs, and very-low-density lipoprotein (VLDL)) [35]. Identification of individual lipid species within lipoprotein subclasses is difficult using NMR. As lipid composition of various lipoprotein subclasses varies considerably, NMR has been applied to measure lipid content in lipoproteins to examine their relationship with CVDs [36, 37] and to determine their genetic determinants [38–42].

MS, on the other hand, provides higher resolution of molecular composition of lipidome [reviewed in 31–34]. MS is either coupled with prior chromatographic separation such as gas chromatography (GC–MS) and liquid chromatography (LC–MS), or involve direct infusion of lipid extract (shotgun lipidomics). LC–MS which provides excellent separation efficiency, high sensitivity and strong specificity, is one of the most important and widely used methods for lipidomics research (Table 1, Supplementary Table 2). Liquid chromatography separates lipids based on their physiochemical properties, i.e., polar head group, carbon chain length, number of double bonds. After chromatographic separation, the isolated lipids are ionized that are detected using a mass analyser [31]. GC–MS provides limited coverage of the lipidome and hence, is restricted to studies focused on specific lipid classes and fatty acids quantifications [43–45]. In recent times, shotgun lipidomics has gain popularity due to its relative simplicity of operation and short run times to quantify hundreds of lipids [32–34] and has been applied in several large-scale studies [12, 46, 47]. Shotgun lipidomics technology directly infuses lipids extract into an electrospray ionization mass spectrometer for the detection of lipids, without chromatographic separation. Shotgun lipidomics has lower sensitivity than LC–MS and hence many of the low abundant lipid species are not captured in shotgun lipidomic approaches.

**Table 1:** Major findings of lipidomics-based epidemiological studies of CVDs

Study Reference, Cohort	Platform	Samples	Lipids analysed
*Ceramides as prognostic markers*			
Sigruener et al. [50], LURIC study	Shotgun MS	3316 (768 incident CVD; 484 mortality)	38 PCs, 15 LPCs, 30 PC Os, 31 PEs, 24 PE Os, 33 SMs, 7 Cers
Cheng et al. [51], ATHEROREMO-IVUS	LC–MS	581 (underwent angiography)	8 lipids (CEs and Cers) identified in LURIC study
Laaksonen et al. [52], Corogene, SPUM-ACS, BECAC	LC–MS	3377 (mostly CVD patients)	4 Cers (C16:0, C18:0, C24:0, C24:1)
Havulinna et al. [54], FINRISK2002	LC–MS	8101 healthy subjects (813 incident MACE)	4 Cers (C16:0, C18:0, C24:0, C24:1)
Wang et al. [53], PREDIMED	LC–MS	1017 (230 incident cases; 787 random samples)	4 Cers (C16:0, C22:0, C24:0, C24:1)
Anroedh et al. [46], ATHEROREMO-IVUS	Shotgun MS	581 (underwent angiography)	10 lipids (CEs and Cers) identified in LURIC study
Alshehry et al. [55], ADVANCE	Targeted LC–MS	3779 (case–control)	Lipidome-wide (310 lipid species)
Paynter et al. [56], WHI, PREDIMED	LC–MS	944 (472 incident CHD; 472 controls); 627 (312 incident CHD; 315 controls)	Lipidome-wide (217 lipids)
Poss et al. [57], Utah population	LC–MS/MS	674 (462 CAD patients; 212 controls)	32 Sphingolipids
*Opposite effects of MUFA and PUFA containing phospholipids*			
Sigruener et al. [50], LURIC study	Shotgun MS	3316 (768 incident CVD; 484 mortality)	38 PCs, 15 LPCs, 30 PC Os, 31 PEs, 24 PE Os, 33 SMs, 7 Cers
Alshehry et al. [55], ADVANCE	Targeted LC–MS	3779 (case–control)	Lipidome-wide (310 lipid species)
Wang et al. [69], PREDIMED	LC–MS	1017 (230 incident CVD; 787 random samples)	Lipidome-wide (200 lipids)
Wurtz et al. [36], FINRISK, SABRE, BWHHS	Targeted NMR metabolomics	7256 (800 CVD events); 2622 (573 CVD events); 3563 (368 CVD events)	14 lipoprotein subclasses and fatty acid composition
Razquin et al. [68], PREDIMED	LC–MS	983 (case–control)	Lipidome-wide (202 lipids)
Mundra et al. [66], LIPID, ADVANCE	LC–MS	5991; 3779	Lipidome-wide (342 lipids)
*TG species and risk of CVDs*			
Fernandez et al. [47], MDC study	Shotgun MS	427 (211 incident CVD; 216 controls)	85 lipids (TGs, DGs, CEs, SMs, PC Os, LPCs, PEs, PE Os)
Stegemann et al. [65], Bruneck study	Shotgun MS	685 (90 incident CVD)	135 lipids (PCs, LPCs, CEs, SMs, PSs, PEs, LPEs, TGs)
Alshehry et al. [55], ADVANCE	Targeted LC–MS	3779 (case–control)	Lipidome-wide (310 lipid species)
Razquin et al. [68], PREDIMED	LC–MS	983 (case–control)	Lipidome-wide (202 lipids)
Wang et al. [69], PREDIMED	LC–MS	1017 (230 incident CVD; 787 random samples)	Lipidome-wide (200 lipids)

*CAD* coronary artery disease, *CE* cholesteryl ester, *Cer* ceramide, *CHD* coronary heart disease, *DG* diacylglyceride, *LPC* lysophosphatidylcholine, *LPE* lysophosphatidylethanolamine, *LC–MS* liquid chromatography–mass spectrometry, *MACE* major adverse cardiovascular event, *MS* mass spectrometry, *NMR* nuclear magnetic resonance, *PC* phosphatidylcholine, *PC O* phosphatidylcholine–ether, *PE* phosphatidylethanolamine, *PE O* phosphatidylethanolamine–ether, *SM* sphingomyelin, *TG* triacyglycerol

## Lipidomics in CVD risk prediction

With the advances in high-throughput lipidomics technologies, several studies were undertaken to perform in-depth examination of role of distinct lipid species in CVDs. The studies and their major findings are described in Supplementary Table 1. Taken together, findings from these studies suggest: (1) ceramides as prognostic markers for CVDs, (2) opposite effects of saturated or MUFA containing lipids and PUFA containing lipids on the risk of future CVD events or death, and (3) distinct role of TG species based on carbon content (Table 1).

### Ceramides as prognostic markers for CVDs

Though first regarded as inert components of cell membrane, sphingolipids have emerged as important bioactive molecules owing to their wide-range of biological functions. Of particular note is the link between ceramides and CVDs mediated through atherosclerotic processes by promoting LDL infiltration into blood vessel wall, aggregation of LDL in arterial plaque and accumulation of cholesterol in macrophages [48, 49]. Findings from in vitro and in vivo animal studies have also supported their role in cardiometabolic disorders including atherosclerosis, and heart failure [27]. In this regard, lipidomics of human plasma in large cohorts not only reinforced the role of ceramides in CVD manifestations, but has also pointed to the distinct ceramide species which are independent predictors of future CVD events or death.

Relationship between distinct ceramide species, particularly Cer(d18:1/16:0), Cer(d18:1/18:0) and Cer(d18:1/24:1), and CVD mortality was first suggested by the LURIC study [50], which was supported by report from ATHEROREMO-IVUS study [51]. This led to a growing interest in these ceramide species among the researchers and several studies focusing on them added to the supporting evidences of their relationship with secondary CVD outcomes [46, 52, 53] and future CVD events in healthy individuals [54]. The findings were also corroborated by lipidome-wide studies [55–58] and a large meta-analysis of seven cohort studies with over 29,800 individuals [59]. On the contrary, a recent study by Seah et al. that explored association of 79 sphingolipid species with CVDs in a Chinese ethnic population did not support the role of ceramides in CVD [60]. Although reasons such as difference in quantification methods and statistical power could not be ruled out, this study may point to the population-specific effect of lipid species on CVD risk. The meta-analysis by Mantovani et al. also suggested that associations may be stronger for ceramides with long acyl chain and for those with unsaturated acyl chain [59]. On the similar lines, Lemaitre earlier showed associations of higher plasma levels of shorter sphingolipids (C16 acyl chain) with increased risk of heart failure, whereas higher levels of longer sphingolipids (C20-24 acyl chains) with decreased risk of heart failure [61].

Clinical utility of prediction scores based on ceramide species has been proposed by several independent studies (Table 2). Laaksonen et al. [52] showed that CERT score based on Cer(d18:1/16:0), Cer(d18:1/18:0) and Cer(d18:1/24:1) and their ratios with Cer(d18:1/24:0) predict cardiovascular death in patients with stable CAD and acute coronary syndromes beyond LDL-C. It was further shown that the prognostic value of CERT score could be improved by adding phosphatidylcholine (PC) species [62]. Subsequently, the predictive value of ceramide-based scores have been repeatedly confirmed by many independent studies including the Framingham Heart study [63], Mayo clinic study [64] and FINRISK2002 [54]. All these strong evidences provided basis for the ceramide-based clinical test recommended by the Mayo clinic [https://news.mayocliniclabs.com/ceramides-miheart/] to assess risk of adverse clinical outcomes in CAD patients. A recent study showed that in addition to ceramides, sphingomyelin species (SM) could be important predictor of CVD and proposed a new risk score termed as the sphingolipid-inclusive CAD (SIC) risk score which included dihydro-Cer(d18:0/18:0), Cer(d18:1/18:0), Cer(d18:1/22:0), Cer(d18:1/24:0), SM(d18:0/24:1), SM(d18:1/24:0), SM(d18:1/18:0) and sphingosine [57]. Authors showed that the SIC risk score provides strong prediction value and outperform other measures including LDL-C and CERT score. Thus, through lipidomics-based studies, plasma ceramides have emerged as promising new diagnostic or prognostic marker for CVD with clinical application.

**Table 2:** Lipidome-based prediction scores for CVD risk and death

Prediction score/Study	Components	C-statistics
CERT1, Laaksonen et al. [52]	Cer(d18:1/16:0), Cer (d18:1/18:0), Cer (d18:1/24:1), Cer (d18:1/16:0)/Cer(d18:1/24:0), Cer (d18:1/18:0)/Cer(d18:1/24:0), Cer (d18:1/24:1)/Cer(d18:1/24:0)	0.80 for CVD death
CERT2, Hilvo et al. [62]	Cer(d18:1/24:1)/Cer(d18:1/24:0), Cer(d18:1/16:0)/PC 16:0/22:5, Cer(d18:1/16:0)/PC 14:0/22:6, PC 16:0/16:0	0.76 for CVD death
SIC, Poss et al. [57]	Cer(d18:1/18:0), Cer(d18:1/24:0), SM(d18:1/24:0), SM(d18:0/24:1)/SM(d18:1/18:0), Cer(d18:1/18:0)/Cer(d18:1/22:0), Sphingosine	0.79 for risk of CAD
Mundra et al. [66]	PC O-34:2, PC 38:5, PI 38:3, PC O-36:1, GM3(d18:1/16:0), LPI 18:2, PE 38:6	0.65 for CVD events
Alshehry et al. [55]	PC O-36:1, CE 18:0, PE O-36:4, PC 28:0, LPC 20:0, PC 35:4, LPC 18:2	0.70 for CVD events
Alshehry et al. [55]	PC O 36:1, DG 16:0_22:5, SM 34:1, PC O-36:5	0.76 for CVD death

C-statistic is a standard measure of the predictive accuracy of a model)

*SIC* sphingolipid-inclusive CAD risk score, *CER* Ceramide, *DG* diacylglyceride, *GM* monosialated ganglioside, *LPC* lysophosphatidylcholine, *LPI* lysophosphatidylinositol, *PC* phosphatidylcholine, *PCO* phosphatidylcholine-ether, *PE* phosphatidylethanolamine, *PEO* phosphatidylethanolamine-ether, *SM* sphingomyelin

### Opposite effects of MUFA and PUFA containing phospholipids

Many distinct phospholipid species have been consistently identified as risk factor for CVDs in lipidomics-based studies (Supplementary Table 1) [47, 50, 55, 56, 65–69]. Recently, alterations in phospholipids levels in patients with ischemic cardiomyopathy have been shown suggesting the changes in metabolic profiles during progression from ischemic heart disease to ischemic cardiomyopathy [70]. Addition of phospholipids to the base model of traditional risk factors also improved CVD risk prediction (Table 2). Bruneck study showed that addition of a phosphatidylethanolamine species PE 36:5 and two other lipid species to a model including conventional risk factors increased prediction value [65]. Similarly, LIPID study [66] and ADVANCE trial [55] showed that addition of phospholipid species to the traditional risk factors improved prediction of CVD events and mortality (Table 2). Although clinical utility of lysophospholipids has also been suggested by Ganna et al. [67], there have been inconsistent reports on the direction of their effect on CVDs [47, 55, 65].

An interesting observation that emerged from these studies is that the phospholipids have opposite effects on CVD risk based on the degree of unsaturation of their acyl chains. It was first observed in the LURIC study that phospholipids with saturated and monounsaturated fatty acyl chains were positively associated with risk of CVD, while polyunsaturated phospholipids were inversely associated with the CVD risk [50]. Later, several lipidome-wide investigations including ADVANCE trial, LIPID study, PREDIMED trial, WHI study and Bruneck study provided consistent findings (Table 1). On the similar lines, Wurtz et al. showed that MUFAs levels increase cardiovascular risk, while higher omega-6 and omega-3 PUFAs lower the risk [36]. Consistently, network-based analysis of lipidome data in PREDIMED study also showed that the lipid species are clustered based on degree of unsaturation and that the cluster containing phospholipids with more double bonds was associated with decreased risk of CVD [69]. Thus, the lipidomics-based studies have suggested that there are two subgroups of phospholipids based on the degree of unsaturation that have opposite effects on CVD pathophysiology.

### Distinct role of TG species in CVDs

The routine clinical risk assessment quantifies the total mass of triglycerides, however, their contribution to the development of CVD has been debatable as clinical trials of lowering TG with fibrates provided inconsistent results [71, 72], whereas genetic evidence supported the causal role [73]. The apparent inconsistency is not surprising due to the large number of functionally diverse TG species in circulation that have varied effects (opposite directions or different magnitude) on CVD risk, as revealed by high-resolution lipidomics. The MDC study first identified association of five TG species including TG 48:1, TG 48:2, TG 48:3, TG 50:3 and TG 50:4 with adverse CVD outcome, after adjusting for Framingham risk factors [47]. Later, many TG species were found to be associated with CVD risk over a 10-year observation period in the Bruneck study [65], but associations were more pronounced for TGs of lower carbon number and double-bond content (saturated and MUFAs). Similarly, ADVANCE study found inverse association of TG 56:6 with recurrent CVD incidence and CVD mortality [55] whereas PREDIMED trial showed that short TGs were associated with increased risk of CVD [68]. These findings were further supported by the network and cluster analysis of lipidome in PREDIMED trial which showed that saturated TGs cluster consisting mainly of DGs and TGs with saturated fatty acids was associated with increased CVD risk [69]. These studies clearly suggest that abnormalities in different TG molecular species levels could have different pathological consequences, which might not be detected in enzymatic measurement of total triglycerides, as evident from a study that found decrease in a TG species without observed change in total triglycerides [74].

## Genetic regulation of lipidome

Despite the expected influence of dietary intake on the circulatory lipids, contribution of genetic factors in endogenous regulation of lipid metabolism is well recognized. Studies using pedigree information and genetic data have shown that 10−60% of the variation in plasma levels of circulatory lipid species is contributed by the genetic factors, with considerable variation across lipid categories [12, 75–77]. For example, in general, sphingolipids have higher heritability than glycerophospholipids, with ceramides having highest estimated SNP-based heritability (35–40%) and phosphatidylinositols with the least heritability (11–31%) [12]. Interestingly, genetic mechanisms do not regulate all human plasma lipid species belonging to a lipid class in the same way [12, 75, 76], as also observed in mice lipidomics studies [78, 79]. Rather, it seems to depend on the length and degree of unsaturation of the acyl chains. For instance, lipids containing polyunsaturated fatty acids have higher heritability compared to other lipid species [12]. It is also reported that phosphatidylcholine species (PCs) with larger number of carbon atoms have lower heritability estimates, while PCs with a larger number of double bonds have higher heritability [80].

**Fig. 4:**
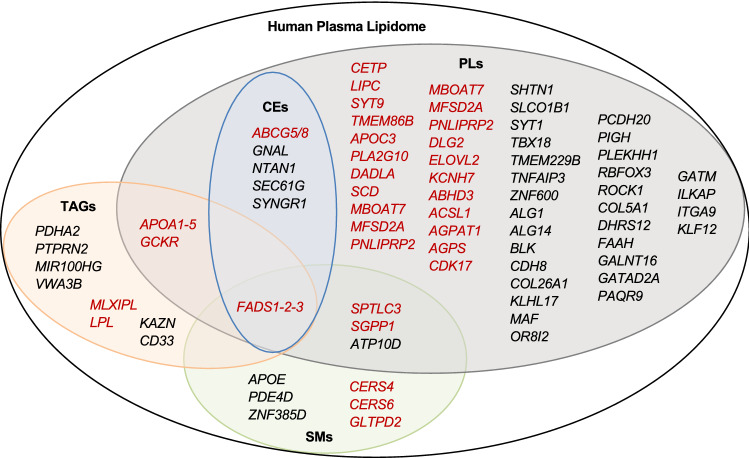
Genetic loci identified in GWAS with lipidome. Genetic loci identified for different lipid classes and their overlap are shown. Genes highlighted in red font have direct role in lipid metabolic pathways. SMs: Sphingomyelins; PLs: Phospholipids; CEs: Cholesteryl esters; TGs: Triacylglycerides

Although over 400 genomic loci are now known to influence the plasma traditional lipid levels [81, 82], their effects on detailed lipid metabolism at molecular levels are not completely known. As epidemiological studies have demonstrated that distinct molecular species (e.g., TGs and PCs) have different or opposite effect on disease outcomes, genetic variants/loci could potentially have different effect on functionally diverse lipid species. To ascertain that, several genome-wide association studies have been performed with individual lipid species (Table 3) [12, 38–45, 83–100]. These studies have not only identified several new loci/genes contributing to lipid metabolism, but also provided novel mechanistic insights to the known loci identified for traditional lipids. The major findings of these GWASs are discussed here. Figure 4 illustrates the genes identified for different lipid categories and their overlap. The list of all the genetic variants reported to be associated in these studies is provided in Supplementary Table 3.

**Table 3:** Genome-wide association studies with lipidome and the identified loci

Study [Ref]	Lipids included	Study population	Loci identified
Gieger et al. [83]	85 sphingolipids, 208 phospholipids	German (*N* = 284)	*FADS*, *LIPC*, *PLEK*
Hicks et al. [84]	33 Sphingolipids	European (*N* = 4400)	*SPTLC3*, *LASS4*, *SGPP1*, *ATP10D*, *FADS1-3*
Illig et al. [85]	PCs, SMs	German (*N* = 1809), British (*N* = 422)	*FADS1*, *ELOVL2*, *PLEKHH1*, *SYNE2*, *SPTLC3*
Lemaitre et al. [43]	n-3 PUFAs (ALA, EPA, DPA, DHA)	European (*N* = 8866)	*FADS1*, *ELOVL2*, *GCKR*
Suhre et al. [86]	Fatty acids, PCs, LPCs, MGs,	German (*N* = 1768), British (*N* = 1052)	*FADS1*, *SCD*, *CYP4A*, *SLCO1B1*, *PDXDC1*, *ELOVL2*
Nicholson et al. [38]	Phospholipids	British (*N* = 211)	None identified for lipids
Kettunen et al. [40]	Phospholipids and CEs content in lipoproteins	Finnish (*N* = 8330)	*PPP1R11, ALB, FCGR2B, ANGPTL3, LPL, APOA1-C3-A4-A5, APOE-C1-C2, LDLR, PCSK9, MLX1PL, CETP, LIPC, PLTP, LIPG, CPT1A, ABCA1, FADS1-2–3, PDXDC1*
Demirkan et al. [87]	PCs and SMs	European (*N* = 4034)	*ABHD3, AGPAT1, ALG1, ALG14, APOA5, APOE-C1-C2-C4, ATP10D, CDH8, CDK17, CNTNAP4, DLG2, ELOVL2, GCKR, ILKAP, ITGA9, KCNH7, KLF12, LASS4, LIPC, LPAR2, OR8I2, PAPD7, PAQR9, PLD2, PCDH20, PDXDC1, PKD2L1, PLEKHH1, PNLIPRP2, SGPP1, SPTLC3, SYT9, ZNF600, FADS1-2-3*
Wu et al. [44]	FAs 16:0, 16:1n-7, 18:0, 18:1n-9	European (*N* = 8961)	*ALG14, LPGAT1, FADS1-2-3, PKD2L1, HIF1AN, GCKR*, rs6722456
Rhee et al. [88]	PCs, TGs, LPCs, SMs, LPEs, CEs, DGs,	American (European ancestry) (*N* = 2076)	*FADS1-2-3, LIPC, GCKR, APOA1/C3/A4/A5, SLCO1B1, SYNE2, PDE4D, SEC61G*, rs6593086, *GNAL, BX18, NTAN1*
Yu et al. [89]	FAs, lysolipids	African American (*N* = 1260)	*PKD2L1*
Shin et al. [90]	FAs, lysolipids	European (*N* = 7824)	*FADS-1-2-3, APOA5, SLCO1B1, LIPC, MBOAT7, SPTLC3*
Ried et al. [91]	Phospholipids	European (*N* = 1809 + 843)	*CPS1, MYL1, MSH4, FNIP2, RABGGTB, SNORD45A, SNORD45B, SNORD45C, C4orf46, PPID, FTFDH, SLC16A9, FADS1-2-3, MIR1908, MIR611, BEST1, RAB3IL1, FEN1, C11orf9, DAGLA, SYNE2, SGPP1, SLC22A4, P4HA2, PDLIM4, SLC22A5, ACADS, CABP1, MLEC, POP5, UNC119B, SPPL3, OASL, SLC22A1, IGF2R, MFSD2A, MYCL1, PDCD6IP, IL3, C12orf75, INTS8, DKFZp68601327*
Draisma et al. [92]	PCs, LPCs, SMs,	European (*N* = 7478 + 1182)	*ABHD3, AGPS, APOE-C1-C2-C4, CERS4, ACSL1,* *CETP, ELOVL2, FADS1-2-3, GATAD2A, GCKR, MFSD2A, PDXDC1, SCD, SGPP1, SPTLC3, TMEM229B, APOA1/C3/A4/A5*
Mozaffarian et al. [93]	Trans FAs from isolated phospholipids	European (*N* = 8013) + non-European (*N* = 1082 + 669 + 657)	*FADS1-2-3*
Kettunen et al. [41]	Lipids content in Lipoproteins, plasma FAs	European (*N* = 24,925)	*PCSK9, SORT1, USF1, GALNT2, APOB, ABCG5, LPA, HMGCR, DNAH11, MLX1PL, ABCA1, LPL, TRIB1, TTC39B, ABO, CILP, ZNF259, SCARB1, LIPC, CETP, LIPG, ANGPTL4, LDLR, APOE, MAFB, PLTP*
Rhee et al. [94]	PCs, TGs, LPCs, SMs, LPEs, CEs, DGs,	European-American FHS (*N* = 2076) + ARIC (*N* = 1528)	None identified for lipids (Exome array)
Yu et al. [95]	FAs, lysolipids	African-American (1361 + 508)	None identified for lipids (LoF analysis)
Hu et al. [45]	MUFAs	Meta-analysis of Chinese and European cohorts (*N* = 3521 + 12,020)	*FADS1-2-3, PKD2L1, GCKR, HIF1AN, LPCAT3*
Long et al. [96]	PCs, LPCs, SMs, Fas	British (*N* = 1960)	*LIPC, APOA5, CERS4, FAAH/CYP4A11, FADS1-2-3, MBOAT7, PDXDC1, PIGH, SCD, SGPP1*
Yousri et al. [97]	PCs, LPCs, SMs, Fas	Qatari (*N* = 614 + 382)	*ACADS, FADS2*
Feofanova et al. [98]	FAs, Lysolipids	European American (*N* = 1552) + African American (*N* = 1872)	*ALMS1P, FADS1-2-3, PKD2L1, SLCO1B1, ELOVL2, PKD2L1, TMC4, LIPC*
Tabassum et al. [12]	PCs, LPCs, PEs, CEs, TGs, SM, Cer	Finnish (*N* = 2045)	*KLHL17, KAZN, ABCG5/8, VWA3B, ZNF385D, SYT1,* *PAQR9, PDHA2, ABLIM2, DDX43, TNFAIP3, SHTN1, COL26A1, PTPRN2, BLK, LPL, CD33, COL5A1, MAF,* *FADS2, APOA5, MIR100HG, DHRS12, SYNE2, LIPC,* *GALNT16, GATM, GLTPD2, RBFOX3, CERS4, ROCK1*
Yazdani et al. [99]	FAs, lysolipids	European American (*N* = 1376)	*MYO1A, PDE4DIP, EGFL8, CCDC154, ZNF211, GPR97, CLEC4C, MAP10, KIAA1755, CD300C*
Demirkan et al. [13]	TGs, PCs, LPCs, PEs, SMs	European (*N* = 5537)	*GCKR, FADS1, APOA, SGPP1, TMEM229B, LIPC, PDXDC1, CETP, CERS4, SPTLC3*
Lotta et al. [100]	PCs, LPCs, SMs	European (*N* = 86,507)	*CERS6*
Coltell et al. [42]	PUFAs	European (N = 426)	*FADS1-2-3*

The genetic loci identified for lipid species in different GWASs are listed. Details of the associations between genetic variants and lipids are provided in Supplementary Table 3

*ALA* α-Linolenic acid, *CE* cholesteryl ester, *Cer* ceramide, *DG* diacylglyceride, *DPA* docosapentaenoic acid, *DHA* docosahexaenoic acid, *EPA* eicosapentaenoic acid, *FA* fatty acid, *LPC* lysophosphatidylcholine, *LPE* lysophosphatidylethanolamine, *MUFA* monounsaturated fatty acid, *PC* phosphatidylcholine, *PC* *O* phosphatidylcholine-ether, *PE* phosphatidylethanolamine, *PE O* phosphatidylethanolamine-ether, *PUFA* polyunsaturated fatty acid, *SM* sphingomyelin, *TG* triacyglycerol

### Sphingolipids

The first genetic investigation of sphingolipids was performed by Geiger et al. in 2008 [83], that included 85 sphingolipids and 284 individuals of the KORA study. The study identified two loci for sphingolipids-*PLEK* and *ANKRD30A* at genome-wide significance that did not stand multiple testing correction. Later, large-scale studies for 33 sphingolipids including over 4400 subjects from 5 diverse European populations identified 7 loci- *ATP10D*, *FADS1-2-3*, *SGPP1*, *CERS4*, *SPTLC3*, *APOE* and *GLTPD2*-*PLD2* [84, 87]. As most of the loci identified by Hicks et al. [84] contains genes encoding enzymes involved in sphingolipids/ceramide synthesis (Fig. 3), and none of the genes involved in ceramide degradation or signaling was identified, authors speculated that the plasma levels of ceramide are primarily regulated by genes involved in ceramide production. Given the prominent roles of these genes in sphingolipid metabolic pathways, association of these genes were subsequently replicated in many studies [12, 85, 92, 95]. Furthermore, new loci were also discovered by studies with larger sample sizes including *PDE4D* and *CERS6* [88, 100]. A recent metabolomics-based GWAS in 7 European cohorts with total sample size of over 7,000 that included 14 sphingomyelins confirmed previous associations of *SPTLC3*, *APOE*, *SGPP1*, *CERS4* with sphingomyelins, but no new locus was identified [13].

From these studies, 6 genes (*SPTLC3*, *CERS4*, *CERS*6, *SGPP1, GLTPD2* and *FADS1-2-3*) with direct role in ceramide metabolism have emerged as prominent regulator of plasma levels of ceramides. The rate of ceramide synthesis is regulated by the first step of de novo pathway, which is catalyzed by serine palmitoyltransferase (SPT). *SPTLC3* codes for a subunit of the SPT complex which catalyses the condensation of serine with palmitoyl-CoA (Fig. 3). The increased expression or activity of SPTLC3 could result in increased ceramide production by increasing the influx of sphinganine in de novo pathway (Fig. 3). Several cis-eQTLs (expression quantitative trait) of *SPTLC3* (GTEx v7) are found to be associated with plasma levels of ceramides and sphingomyelins (Supplementary Table 3), suggesting that genetic effect of *SPTLC3* variants is mediated by regulating the expression of the gene. SPT product sphinganine is then metabolized to dihydroceramides by the addition of different acyl-chains by ceramide synthases (CerS) [101], which is subsequently converted to ceramides and sphingomyelins. CerS also catalyzes the conversion of sphingosine to ceramide in the salvage pathway. Six isoforms of CerS (CerS1-6) exist in humans with different preferences for specific fatty acids. CerS4 has high selectivity towards long acyl chains (C18-C20) while CerS6 has preference for short acyl chains (C14-C16) [102]. Consistently, association of variants in *CERS4* that encode CerS4, with ceramides and SMs containing C18-C20 acyl chains have been identified repeatedly in many studies (SupplementaryTable 3).

*FADS1-2-3* locus encodes three enzymes that regulate desaturation of fatty acids and production of unsaturated fatty acids. Unsaturated ceramides are synthesized by the incorporation of unsaturated fatty acids into sphingosine/sphinganine bases. Association of variants in the *FADS1-2-3* locus with unsaturated ceramides levels suggests crucial role of fatty acid desaturases in generation of unsaturated ceramides and sphingolipid metabolic pathways [84]. On the other hand, *SGPP1* codes for sphingosine-1-phosphate phosphohydrolase 1 that play important role in salvage pathway. SGPP1 belongs to the phosphatase super-family that converts sphingosine-1-phosphate to sphingosine that is readily metabolized to ceramide. Enhanced *SGPP1* activity could lead to elevated sphingolipid levels by shifting the stochiometric balance towards sphingosine and ceramide production. Consistently, variants in *SGPP1* have shown association with circulating sphingomyelins, mainly containing C14-C16 and C22-C24 acyl chains. *GLTPD2* codes for glycolipid transfer protein domain-containing protein 2 and has putative role in transfer of ceramide-1-phosphate. Thus, findings from the major GWASs on sphingolipids suggest that the plasma levels of ceramides and sphingomyelins are primarily driven by the genes involved in sphingolipid metabolism, particularly ceramide biosynthesis (Fig. 3).

### Glycerolipids

Only a few GWASs have included molecular TG species [12, 13, 88, 94]. The study by Rhee et al. in over 2,000 participants from Framingham Heart Study (FHS) included 46 TG species and revealed association of *FADS1-2-3*, *GCKR* and *APOA1-5* loci with several TG species [88]. These genes with direct role in triglyceride metabolism were subsequently replicated in other GWASs [12, 94]. The lead *GCKR* variant rs1260326, a missense variant (L446P) associated with TG species, is established as the likely causal variant through functional studies [103, 104]. In another study by Rhee et al. that focused only on the rare variants, no additional variants for TGs could be identified [94]. However, recent studies have suggested association of common variants at new loci for TGs. Our previous study suggested new signals for TGs at genetic variants in or near *KAZN*, *VWA3B*, *ABLIM2*, *PDHA2*, *PTPRN2*, *LPL*, *APOA5*, *CD33* and *MIR100HG* at genome-wide significance that did not remain significant after multiple testing correction [12]. Also, Demirkan et al. identified association between a new locus *MLXIPL* and TGs (TG 48:1 and TG 50:1) [13].

Interestingly, similar to epidemiological finding that different TG species have different effect on CVD risk, findings from the genetic studies revealed different patterns of association of TG species with genetic variants. *GCKR* demonstrated a stronger association with TGs of relatively lower carbon content (TG 48:2, TG 48:3, TG 50:3, TG 50:2, TG 50:4) [13, 88], while *APOA1/A5* and *LPL* have stronger effect on medium length TG species (TG 54:4, TG 52:3, TG 52:4) [12, 88]. On the other hand, *FADS1-2-3* associate with TGs in a fatty acid saturation specific manner, with the direction of effect differed at the extremes of TG carbon content, and strongest association with TGs of relatively higher carbon and double bond content such as TG 58:10 and TG 58:11 [13, 88]. Such a pattern of association was also observed in one of the loci identified in GWASs for enzymatically measured triglycerides-*CILP2* that had different effect sizes across different TG species and was mainly associated with the unsaturated TGs [13]. These findings suggest that genes involved in TG metabolism have species-specific effect that apparently depend on the length of acyl chains. Further light on this was provided by our previous study which showed that a genetic variant at *LPL* locus (rs11570891) increases the expression and enzymatic activity of LPL [12]. We further showed that the increased LPL enzymatic activity had stronger effect on medium length TGs than other TGs. Taken together, GWAS findings suggest that genetic regulation of TGs is determined by their carbon content and degree of unsaturation and further reinforce that such effects might not be detected by enzymatic measurement of total triglycerides.

### Phospholipids

A number of genetic loci have been associated with plasma levels of distinct phospholipids species, including genes with direct role in phospholipid metabolism (Table 3; Fig. 4). In the KORA study that included 208 phospholipid species, Geiger et al. [83] identified association of phospholipids with *FADS1-2-3* and *LIPC*. Later, Illig et al. [85] identified five loci for phospholipids-*FADS1-2-3*, *ELOVL2*, *PLEKHH1*, *SYNE2* and *SPTLC3* in a larger dataset. Further, a comprehensive genetic investigation of phospholipids with 57 PCs, 20 lyso PCs, 27 PEs, 15 plasmalogens in over 4000 samples identified 25 loci at genome-wide significance [87]. In the pathway analysis, 13 genes (*KCNH7*, *AGPAT1*, *PNLIPRP2*, *SYT9*, *FADS2*, *DAGLA*, *DLG2*, *APOA1*, *APOC3*, *ELOVL2*, *CDK17*, *LIPC* and *PLA2G10*) located in 11 loci from the 25 loci were mapped to the glycerophospholipid metabolism pathway [87]. Several additional loci for phospholipids were discovered as illustrated in Fig. 4 and listed in Supplementary Table 3. Here we discuss two examples that highlight the potential of lipidomics in identifying new lipid modifying genes and providing mechanistic insights to the known lipid loci.

*MBOAT7* encodes a lysophosphatidylinositol acyltransferase that incorporates arachidonic acid (C20:4) into lysophosphatidylinositol (LPI) to generate phosphatidylinositols (PI) [105]. The activity of MBOAT7 regulates the levels of free arachidonic acid and its availability for eicosanoid production which mediates pro-inflammatory signalling [106]. Consistent with its biochemical function, Shin et al. identified association of variants in *MBOAT7* with the ratio of arachidonate (20:4n6) to 1-arachidonoylglycerophophoinositol [90]. Later, its association with PI species was confirmed in other studies [12, 96]. *MBOAT7* variants also increase the susceptibility to liver disorders including liver cirrhosis and non-alcoholic fatty liver disease (NAFLD) by inducing a reduction in its expression in liver [107–109]. Notably, our previous study also suggested association of *MBOAT7* variant with venous thromboembolism [12]. The example of *MBOAT7* further exemplifies that lipidome-based GWAS could identify new genes with prominent role in lipid metabolism that could not be detected through GWAS of traditional lipids.

Fatty acid desaturase (*FADS*) gene cluster has been consistently reported to be associated with omega-3 and omega-6 fatty acids levels with inverse effects on different PUFAs [43, 44, 83, 87]. The *FADS* gene cluster contains genes coding for three key enzymes in PUFA metabolism-FADS1 (delta-5 desaturase), FADS2 (delta-6 desaturase) and FADS3 (delta-9 desaturase). The delta-6 desaturation by FADS2 generates gamma-linolenic acid (C18:3 n-6) and stearidonic acid (C18:4 n-3) from linoleic acid (C18:2 n-6) and alpha-linolenic acid (C18:3 n-3) respectively, that by elongation yield dihomo-gamma-linolenic acid (C20:3 n-6) and eicosatetraenoic acid (C20:4 n-3) [110]. Further, delta-5 desaturation of dihomo-gamma-linolenic acid by FADS1 generates arachidonic acid (C20:4 n-6) and eicosapentaenoic acid (C20:5 n-3). Genetic variants in *FADS1* and *FADS2* genes are associated with the increased levels of phospholipids with three or less double bonds while with the decreased levels of phospholipids with four or more double bonds [12, 83]. We previously showed that a variant in *FADS2* increases *FADS2* expression while reduces the expression of *FADS1* that explain the inverse relationship of *FADS2* variants with lipids containing different polyunsatureated fatty acids (PUFAs) [12]. The association of *FADS1-2-3* locus with the reduced levels of lipids containing arachidonic acid may also explain its assocition with reduced risk of atherosclerotic CVD outcomes-peripheral artery disesae (PAD) and aterial embolism and thrombosis [12]. The example of *FADS1-2-3* along with other known lipid genes such as *LPL*, *GCKR* (discussed above) highlights how detailed lipidomic profile could provide the mechanistic understanding of effects of well-established lipid loci.

### Sterols

Sterol lipids including CEs are not represented well in the GWASs with lipidome or metabolome, and only two studies have reported association of CEs with genetic variants [12, 88]. Rhee et al. [88] identified four loci associated with CE species- *FADS1-2-3*, *GNAL*, *NTAN1* and *SEC61G*. We found previously association of three loci with CEs-*ABCG5/8*, *FADS2* and *SYNGR1. ABCG5/G8* codes for ABC cholesterol transporters G5 and G8, that have been associated with total cholesterol, LDL-C and cholesterol esters in LDL. However, our study revealed a novel association of *ABCG5/G8* variant with a specific CE species- CE 20:2;0. All of the identified loci for CEs overlap with the loci associated with phospholipids (Fig. 4).

## Discussion

It is apparent from epidemiological and genetic studies that lipidomics has great potential in revealing new biology not captured by traditional lipids and lipoprotein measurements. Lipid species measurements, like other intermediate phenotypes, increases statistical power to detect genetic associations and hence provide opportunity to discover new lipid loci [12, 111]. In an analysis with over 500 known genetic variants for traditional lipids, we previously showed that as compared to traditional lipids, associations with detailed molecular lipids are several orders of magnitudes stronger for the variants in or near genes involved in lipid metabolism such as *FADS1-2-3*, *LIPC*, *ABCG5/8*, *SGPP1*, *SPTLC3* [12]. This demonstrates the prospects of lipidomics in identifying lipid-modulating variants, particularly the ones with direct role in lipid metabolism. Consistently, GWASs with distinct lipid species discovered many new genes with direct role in lipid metabolic pathways and provided new insights into the genomic loci associated with traditional lipids. For instance, detailed TG profiles revealed that the total triglycerides associated loci such as *GCKR*, *FADS1-2-3*, *LPL*, *APOA5* drive association of distinct TG species depending on number of carbon atoms and degree of unsaturation, as discussed above.

### Integrating lipidomics and genomics: opportunities beyond GWAS

**Fig. 5:**
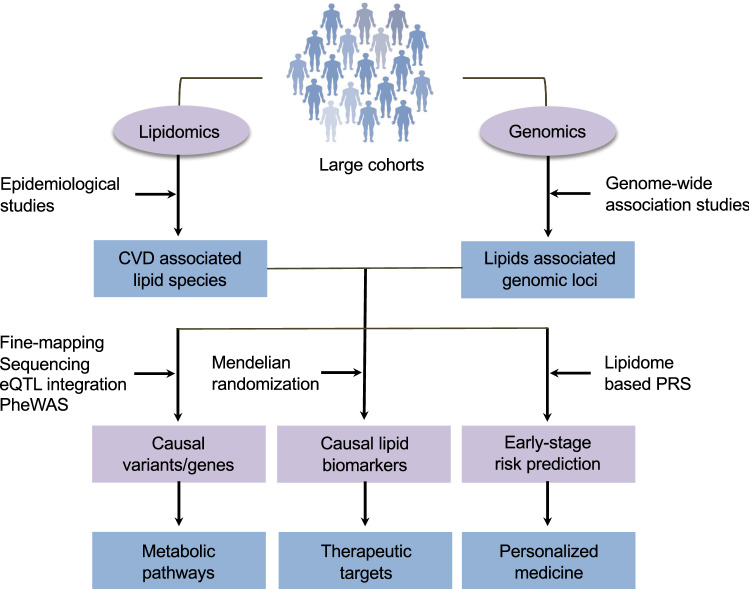
Approaches to move beyond GWAS. New opportunities and prospects of application of genomics to translate findings from lipidomics to develop better predictive and preventive strategies are illustrated

After the success of the GWASs in identifying new genomic loci associated with lipid species, one of the next challenges is to translate these findings towards predictive and personalized medicine. Emerging tools in genomics provides many new opportunities in this direction [112]. This is demonstrated by the success of genetic studies guiding the development of antibodies targeting PCSK9 (proprotein convertase subtilisin/kexin type 9) to treat hyperlipidemia and CVD [113]. Here we discuss approaches that could be used to translate the statistical associations identified in epidemiological studies and GWASs to biological understanding, drug target identification and disease risk characterization (Fig. 5).

### Inferring causality towards drug targets development

Epidemiological studies have provided strong evidence of association of several distinct lipid species with CVD outcomes. However, it cannot be inferred from simple observational studies which of these associated lipid species have causal effect on CVDs. Nonetheless, genomics provides an alternative approach to infer causality using the intrinsic properties of the genome, i.e., the random assortment of alleles at conception, in a statistical framework referred as Mendelian randomization (MR) [114–116]. MR is a routinely used genetic tool in observational studies which uses genetic variants as proxies for exposure variable (risk factors) to infer whether the increased or decreased risk factor causes the disease [41, 117, 118]. First described in 1986 [119], many methods and approaches in MR analysis have been developed that allow use of GWAS summary statistic data for risk factor and disease of interest, either from one dataset (one-sample MR) or two datasets (two-sample MR) [114]. Development of two-sample MR methods using the existing and publicly available GWAS summary data has made MR analysis increasingly easy and popular.

With the improved understanding of the genetic architecture of lipidome and availability of large-scale GWAS summary statistics data both for lipidome and CVD, MR provides an excellent avenue to infer the causal role of the CVD associated lipid species. Efforts at this front have been limited so far, most likely due to the highly correlated nature of the high-dimensional lipidome profiles. Ganna et al. [67] used summary stats from CARDIoGRAMplusC4D to reveal causal effect of MG 18:2 on CHD risk. On the other hand, a recent study performed a MR analysis to investigate the causal relationship between PC 38:3 and P wave duration (PWD, an indicator of atrial conduction) [120]. The study found that PC 38:3 and PWD association is not causal and is mediated by BMI. Larger efforts are required to highlight the lipid species with therapeutic potential from the large number of associations for follow-up investigations, as exhibited in a recent MR analysis [121]. The study performed GWAS on untargeted plasma metabolome with  ~ 11,000 metabolites in  ~ 10,000 individuals and used two-sample MR approach to assess the causal effect of both identified and unidentified metabolites on 45 common diseases. The study provided evidence of causal effect of 31 metabolites on at least one of the 5 diseases- CHD, schizophrenia, bipolar disorder, rheumatoid arthritis, primary sclerosing cholangitis. Among the causal metabolites, 19 metabolites were causal for coronary heart disease and 6 of which were found to be associated with incident CHD. Recent development of multivariate MR methods provides a powerful tool to incorporate high-dimensional data like lipidome profiles in MR analysis [122–124]. Application of these approaches to infer the causality of CVD associated lipid species is one of the ways to move forward in the direction of drug target development.

### Refining GWAS signals to causal variants to reveal metabolic networks

Although GWASs have been successful in identifying new genomic loci associated with lipid species, the associated variant, in most cases, does not cause the trait or disease itself but serve as surrogate for neighbouring SNPs in a large genomic region that are in linkage disequilibrium (LD) with it. Because of the complex LD patterns among the SNPs, pinpointing causal variants from the associated variants is a challenging task. However, statistical fine-mapping approaches allow the refinement of the trait-associated regions to identify genetic variants with likely causal influence on the trait [125, 126]. The fine-mapping approach in lipidomics could not only help in refining the genomic loci but the multidimensional association data may also help in highlighting the metabolite-specific effects and hence reveal new metabolic networks. For instance, Gallois et al. [127] performed fine-mapping of *LIPC* region which suggested that there are at least three distinct sites with metabolite-specific variants within the gene. The study showed that large HDL and triglyceride in lipoproteins are influenced by all the three sites in *LIPC.* But interestingly, intermediate density lipoproteins (IDLs) and fatty acids are mostly influenced by two sites and very small VLDL (very low-density lipoprotein) are influenced by only one of sites in the gene [127]. On the similar note, earlier Tukiainen et al. [111] showed that *LIPC* region has opposite associations between the lipid measures of larger and smaller HDL particles.

Another commonly used approach is targeted sequencing of the trait-associated region that allow identification of rare coding or loss-of-function (LoF) variants with putative causal effects in the region [128–132]. Exome or whole genome sequencing allow the identification of full spectrum of variants, including rare and loss-of-function variants that may have direct functional effects than the common variants [99, 133, 134]. Due to the limited sample sizes, the sequencing efforts have been so far less successful in lipidomics and only a few rare or LoF variants influencing lipid levels could be discovered [96–99]. Long et al. identified seven rare variants in four genes (*ACADS*, *CRAT*, *DMGDH*, *ETFDH*) involved in fatty acid metabolism [96]. Thus, larger sequencing efforts are required to better understand the potential role of rare coding variants involved in lipidomic variation.

Further insight to the putative causal variant could also be provided by integrating GWAS results with other “omics” data such as transcriptome, proteome and epigenome. About 80% of the genetic variants identified by the GWASs lie in the non-coding regions, thus exploring the association with gene expression levels (eQTLs), protein levels (pQTLs) and epigenetic changes such as DNA methylation in relevant tissues could provide information about the biological effects of the variants and putative causal genes. Also, utilizing the phenome-wide association (PheWAS) data for thousands of clinical outcomes from biobanks such as UK Biobank (https://www.ukbiobank.ac.uk/) and FinnGen (https://www.finngen.fi/en) could further provide mechanistic insights. To facilitate the efforts in this direction, colocalization tools have been developed in recent years that can integrate multi-omics datasets such as GWAS, eQTLs, pQTLs and PheWAS data using statistical methods [135–137]. The colocalization analysis by Franceschini et al. provided evidence for the role of novel genes in the subclinical measures such as carotid intima-media thickness (cIMT) and carotid plaque formation and provided insights into the regulatory mechanisms linking atherosclerosis and clinical outcomes [137].

### Predicting CVD risk using lipidome-based genetic risk scores

Early prediction and prevention could greatly reduce the enormous socio-economic burden of the CVDs [138]. The intensity of risk management regime is generally guided by the risk estimates of the individuals [139], which are not always precise [140]. Moreover, existing clinical risk assessment tools, that typically include traditional plasma lipids, identify individuals with high CVD risk at a stage when atherosclerotic events have already developed. Polygenic risk scores (PRS), a weighted sum of the number of risk alleles carried by an individual, has shown potential in early prediction, but currently have limited clinical utility [141–144]. Thus, the quest for better and early-stage prediction scores to maximize the benefits of risk management has been the focus of the CVD research, but have provided limited success so far [145, 146].

One of the challenges in early CVD detection and prevention is the heterogeneity owing to the diverse pathological conditions that are preceded by atherosclerotic and metabolic events developing at young age [147], resulting in different CVD subtypes. Hence, individuals’ predisposition to different subtypes is influenced by multiple independent risk factors that need to be understood and incorporated in prediction algorithms to guide appropriate and personalized interventions. Traditional lipid profiling that measures HDL-C, LDL-C, triglycerides and total cholesterol, does not reflect precise molecular perturbations in lipid metabolism underlying CVD subtypes. Moreover, a PRS based on genetic loci for CVD represents a combination of genetic risk factors acting through different pathways, whose roles may vary in different CVD subtypes. In such scenario, individuals would respond very differently to the same risk management strategies. As revealed by genetic studies of lipidome that many genomic loci have lipid species specific effects, integrating information of genetic variants of lipidomic measures in PRS algorithms could provide more specific and sensitive CVD risk stratification than those based on CVD variants. Our proposition is that the risk prediction model should incorporate information on perturbations in individual's lipidome profile and their genetic determinants. Thus, the next challenge is to develop predictive tools to incorporate the genetic data on high-dimensional lipidome profiles.

## Conclusion

The increasing global burden of CVDs highlights the pressing need for better personalized prediction and prevention strategies. One key step is to open new therapeutic opportunities by understanding the causal roles of lipid metabolism at molecular lipid species/sub-species resolution in heterogenous CVDs etiologies and their regulation by genetic and lifestyle factors. To this end, the technological advances in lipidomics and other omics technologies have led to a tremendous progress in the CVD research field in last two decades. Lipidomics has not only provided a closer look at the lipid metabolic perturbations in CVDs, but has improved our understanding of the genetic control of lipid metabolism. Further improvement in technologies will continue to improve our understanding of CVDs. However, the ultimate goal of a personalized translational research is to find the right intervention (target biomarker) for right individual (CVD subtype) at the right time (at early stage). Integrating the emerging genomics tools with the high-dimensional lipidome holds a great potential in moving towards this goal. We discussed some of the commonly used approaches in translational research that could be employed in lipidomics-based studies, however further advancements in statistical and computational tools would be required to deal with the high-dimensional and correlated structure of the lipidome profiles.

### Supplementary Information

Below is the link to the electronic supplementary material.

Supplementary file1 (XLSX 63 KB)

## Data Availability

Not applicable.
